# Diamond Chemistry: Advances and Perspectives

**DOI:** 10.1002/anie.202418683

**Published:** 2025-05-23

**Authors:** Nianjun Yang, Anke Krueger, Robert J. Hamers

**Affiliations:** ^1^ Department of Chemistry Hasselt University Agoralaan 1 Diepenbeek 3590 Belgium; ^2^ IMO‐IMOMEC Hasselt University Wetenschapspark 1 Diepenbeek 70569 Belgium; ^3^ Institute of Organic Chemistry University of Stuttgart 70569 Stuttgart Germany; ^4^ Center for Integrated Quantum Science and Technology (IQAST) University of Stuttgart 70569 Stuttgart Germany; ^5^ Department of Chemistry University of Wisconsin─Madison Madison WI 53706 USA

**Keywords:** Catalysis, Diamond chemistry, Energy, Quantum science, Sensing

## Abstract

Diamond as a material has many unique properties. Its high optical dispersion, extraordinarily high mechanical strength, and unparalleled thermal conductivity have long made it a material of interest for applications such as high‐temperature electronics and as wear‐resistance coatings. More recently, diamond has emerged as a material with a wide range of applications in chemistry and biology. The high intrinsic stability of diamond, coupled with the ability to modify diamond surfaces with a wide range of inorganic, organic, and biological species via highly stable covalent linkages, provides a wealth of opportunity to couple diamond's chemical properties with its extraordinary physical properties. The practical utility of diamond has been greatly expanded in recent years through dramatic advances in the ability to produce diamond in bulk, thin film, and nanoparticle form, with controlled doping and purity at modest cost. These advances, together with diamond's highly stable and tunable surface chemistry with versatility of physical structure enable a wide range of emerging applications of interest to chemists, including quantum science, biomedicine, energy storage, and catalysis. Yet, to fully exploit the unique properties of diamond, some formidable chemical challenges lie ahead.

We begin by reviewing some of the features of diamond that are of particular importance to the chemistry community. We aim to highlight some of the important applications where diamond chemistry plays a key role, identify some of the key observations, and outline some of the future directions and opportunities for diamond in the chemical world.

## Graphical abstract

In their review, Yang, Krueger, and Hamers highlight recent advances in diamonds that impact chemical scientists. Advances in growth of diamonds have yielded improved quality and lower cost, while new and emerging applications in areas such as energy storage, photo‐ and electro‐catalysis, sensing, and quantum science have expanded applications.

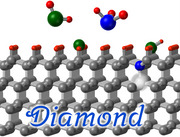

## 1 Introduction to Diamond Chemistry

This section discusses the general properties of undoped and doped diamond, especially its extraordinarily high stability, well‐organized sp^3^ carbon structure, and highly versatile surface chemistry.

### 1.1 Unique Features of Diamond Materials

Diamond has many unusual properties that make it especially technologically useful. As a wide‐bandgap semiconductor, diamond can be intentionally doped, making it useful for electronic applications that require operation under unusual conditions such as high temperature. Unlike most other semiconductor materials, diamond is chemically stable even in harsh aqueous chemical environments, including high and low pH, the presence of high salt concentrations, and electrochemical and biological environments.^[^ ^1^, ^2^, ^3^ ^]^ The high chemical stability of diamond makes it uniquely suited to couple semiconductor properties to chemical properties, thereby opening up new applications such as chemical and biological sensing.^[^ ^1^, ^4^ ^]^ Recently, diamond has gained attention because certain kinds of defects in diamond have unusual spin coherence properties, enabling optical readout of electron spin states. Making use of diamond's quantum properties enables a wealth of new applications in quantum computation, memory, and sensing.^[^ ^5^ ^]^ Diamond's extraordinary chemical and physical stability enables its use in extreme environments, including over a wide range of electrochemical potentials, under highly acidic/basic conditions, and in complex biological environments, as well as at extremes of temperature. These complement diamond's mechanical and optical properties, which, due to space limitations, are not discussed in this minireview.

### 1.2 General Chemical Applications of Diamond Materials

Much of the interest in diamond is motivated by the fact that while diamond has many unique physical properties, many of the applications of diamond are impacted by or enabled by diamond's chemical properties. Here, we briefly highlight some of these applications where diamond chemistry is particularly important.

#### 1.2.1 Chemical/Biochemical Sensing

Diamond films and powder‐based electrodes have been widely employed for chemical and biochemical sensing of various targets via electrochemical means. These applications frequently take advantage of the ability to dope diamond with boron, making the diamond electrically conductive. Diamond has an especially wide window of electrochemical stability in water and has a low efficiency for oxidizing water or reducing H^+^ to H_2_, reactions that frequently give rise to background currents that limit sensitivity using other electrodes.^[^ ^6^ ^]^ In contrast, the low background current and a wide window of electrochemical stability of boron‐doped diamond (BDD) electrodes enable simultaneous and sensitive monitoring of both individual and multiple substances by means of electrochemical approaches. Moreover, BDD electrodes can be employed for electroanalysis even in harsh situations due to their high level of chemical inertness and stability. Analytes of interest range from metal ions^[^ ^7^, ^8^ ^]^ to complex organic mixtures.^[^ ^9^ ^]^ In some cases, simultaneous detection of several analytes has been realized, such as electrochemical determination of herbicides,^[^ ^10^ ^]^ multiple ions in an ethanolic medium,^[^ ^11^ ^]^ simultaneous detection of acetaminophen, caffeine, and carisoprodol in pharmaceutical formulations,^[^ ^12^ ^]^ and amino‐substituted aromatic compounds.^[^ ^13^ ^]^ Diamond electrodes have been further modified with nanometer‐sized graphite, metal nanoparticles and applied as electrochemical sensors for the detection of such targets.^[^ ^14^, ^15^, ^16^, ^17^ ^]^

Diamond materials possess the advantages of excellent biocompatibility, chemical inertness, nontoxicity, and cell attachment properties. Diamond can be used as a fluorescent tag either by chemical modification to link fluorescent ligands to its surfaces or by modifying the diamond lattice itself to place highly luminescent color centers, such as nitrogen vacancy (NV) centers, inside the diamond lattice where they are chemically protected from the outside environment. Consequently, diamond nanoparticles have been thus intensively applied for labeling of cells, bioimaging, biosensing, and drug delivery. For example, fluorescent nanodiamonds have been successfully used in biosensing and bioimaging.^[^ ^18^, ^19^ ^]^ Modification of diamond surfaces with chemical structures that provide molecular recognition ability can yield chemical specificity in how diamond interacts with different analytes in complex media (e.g., blood, urine, dialysate), enabling nanoparticles and other nanostructures to be used for labeling of cells, bioimaging, biosensing, and targeted drug delivery. Examples include blood glucose detection,^[^ ^20^ ^]^ determination of universal biomarkers for the influenza virus, M1 protein,^[^ ^21^, ^22^ ^]^ electrochemical identification of the SARS‐CoV‐2 virus,^[^ ^23^ ^]^ and detection of oxidative phosphorylation expression in hepatocellular carcinoma cells with aid of artificial intelligence.^[^ ^24^ ^]^ Optimum utility of diamond in these applications requires control of both the surface and the bulk properties, such as the presence of sp^2^‐hybridized nondiamond carbon, the presence of dopants or other foreign atoms in the diamond lattice, and the composition of carbide‐based electrodes.^[^ ^25^, ^26^, ^27^ ^]^

#### 1.2.2 Diamond Electrodes for Large‐Scale Water Purification and Environmental Remediation/Degradation

In recent years, the use of diamond materials for the removal and decomposition of problematic pollutants has gained momentum.^[^ ^27^ ^]^ Perhaps counter‐intuitively, the slow rates for electrochemical reduction of H^+^ to H_2_ and oxidation of H_2_O to O_2_ represent a unique advantage for diamond by increasing Faradaic efficiency for diamond to induce even more energetic reactions in water. The ability of diamond electrodes to produce hydroxyl radicals and other reactive oxygen species provides a way to achieve water purification and sterilization.^[^ ^28^, ^29^ ^]^

An extensive range of environmental pollutants in wastewater has been degraded by conductive diamonds, i.e., BDD.^[^ ^30^ ^]^ With the assistance of persulfate oxidation, it has been demonstrated that BDD electrodes are effective for the oxidation of organic compounds and cyanide in complex chemical wastewater.^[^ ^31^, ^32^ ^]^ A significant improvement was observed in the degradation efficiency of organics and cyanide in comparison with electrochemical oxidation without persulfate. Furthermore, the energy consumption of electrolysis was also simultaneously reduced by a considerable amount. Meanwhile, nanodiamond electrodes have been applied for removal of toxic metal ions from wastewater.^[^ ^33^ ^]^ Further examples include the removal of antibiotics,^[^ ^34^ ^]^ perfluorinated compounds such as PFOA,^[^ ^35^ ^]^ pain killers,^[^ ^36^ ^]^ organic dyes,^[^ ^37^ ^]^ and residues from the citrus industry.^[^ ^38^ ^]^ The ability of diamond to emit electrons into water when excited with energetic photons is an additional pathway to reactive species that can enhance its ability to be used for the successful removal of problematic compounds.^[^ ^39^, ^40^, ^41^ ^]^

#### 1.2.3 Catalysis and Electrocatalysis

**Figure 1:**
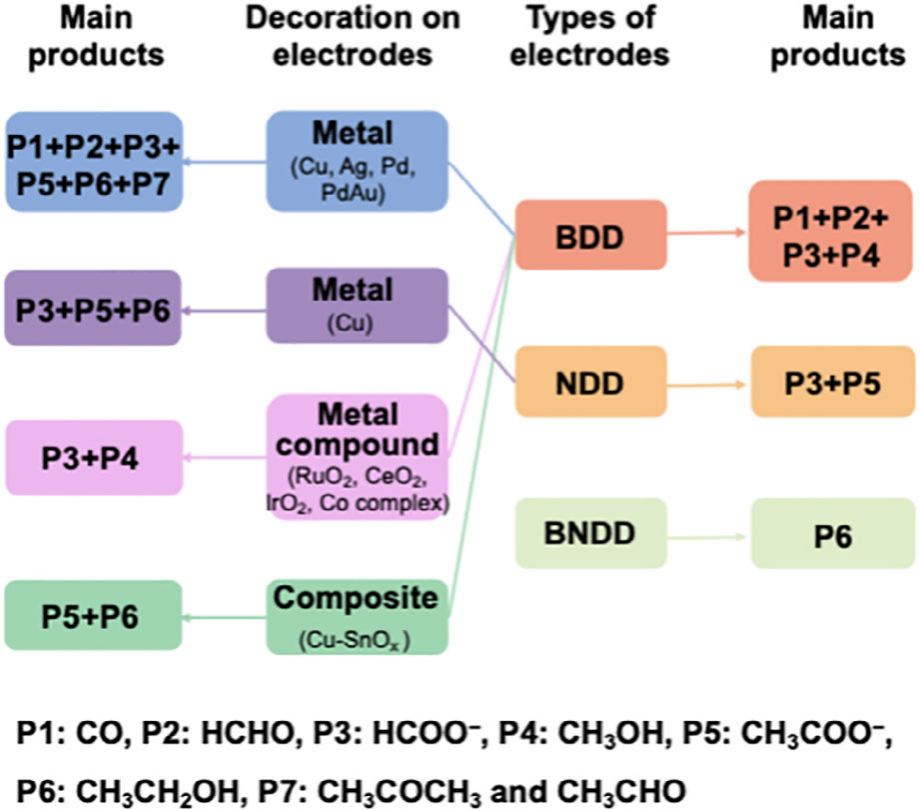
Summary of electrochemical CO_2_ reduction products on different diamond electrodes before and after their decoration with various materials. The terms BDD, NDD, and BNDD stand for boron‐doped diamond, nitrogen‐doped diamond, and B,N co‐doped diamond, respectively.

Diamond's outstanding stability and conductivity allow it to be used as catalyst and/or electrocatalyst material that can be especially beneficial for thermodynamically challenging reactions such as reduction of CO_2_ and N_2_. Diamond's surface chemistry plays a key role. As noted above, diamond exhibits very slow kinetics for H^+^ reduction in water. As a result, the selectivity toward other reactions, especially those that are less thermodynamically favorable, is improved. More recently, diamond electrochemistry has been extended to photochemistry as well, such as the photochemical reduction of CO_2_ into CO^[^ ^39^, ^40^ ^]^ and nitrogen into NH_3_^[^ ^41^ ^]^ in water has been achieved on hydrogen‐terminated diamond electrodes. Meanwhile, electrochemical reduction of CO_2_ (Figure 1) to various C_1_ species (e.g., CO, HCHO, HCOOH) has been achieved using BDD electrodes,^[^ ^2^, ^42^, ^43^, ^44^ ^]^ while C_2_ products (e.g., acetate, CH_3_COO^−^) are preferentially formed on nitrogen‐doped nanodiamond/Si rod arrays—an efficient nonmetallic electrocatalyst.^[^ ^45^, ^46^ ^]^ The electrocatalytic selectivity and efficiency of the CO_2_ reduction reaction on B and N co‐doped diamond (BNDD) electrodes for ethanol (CH_3_CH_2_OH) production were highly enhanced.^[^ ^47^ ^]^ This is due to the synergistic effect of B and N co‐doping on electrocatalytic activity of carbon materials and a fine balance between the N content and H_2_ evolution potential. In these reactions, diamond is especially beneficial under electrochemically reducing conditions, since the competing reaction of H^+^ reduction is particularly facile on metals and most other electrode materials. In these cases, no additional catalysts were added onto these diamond electrode surface.

Although the mechanisms of electrochemical CO_2_ reduction on BDD electrodes in aqueous solution are varied,^[^ ^48^ ^]^ the selectivity of electrochemical CO_2_ reduction on diamond can be further modified by depositing metals (e.g., Pt, Cu) or metal oxides on diamond electrodes. Namely, catalyst‐decorated diamond electrodes have been employed for selective electrochemical CO_2_ reduction. For example, on both Ag‐ and Pd‐coated BDD surfaces, the main reduction products were C_1_ products such as CO and HCOOH (but with varied Faradaic efficiencies). In contrast, on Cu and Pd/Au‐coated surfaces, reduction products including HCOO^−^ and CH_3_COO^−^ have been generated. Of especial interest is that this process has been shown to improve the selectivity on Cu‐coated nitrogen‐doped diamond (NDD) surface—reducing CO_2_ to C_2_ species: ethanol (CH_3_CH_2_OH).^[^ ^45^, ^46^, ^49^, ^50^ ^]^ Meanwhile, it was also demonstrated that both sp^2^ carbon content or the sp^2^/sp^3^ carbon content ratio of BDD as well as the boron doping level affect product selectivity or the Faradaic efficiencies of the generation of different reduction products during electrochemical CO_2_ reduction. An increase of sp^2^ carbon concentration leads to enhanced Faradaic efficiencies to produce hydrogen while decreasing the Faradaic efficiencies to produce C_1_ products (e.g., formic acid, HCOO^−^).^[^ ^51^, ^52^ ^]^

While graphite and other sp^2^‐based carbons have been used as electrode materials for many years, the sp^3^‐hybridization of carbon in diamond provides unique chemistry. It has been reported that boron‐doped, nanostructured diamond can be used as an electrocatalyst for the nitrogen reduction reaction (NRR), providing a high NH_3_ yield rate, high Faradaic efficiency, and stable operation.^[^ ^53^ ^]^ Substitutional boron atoms are illustrated to be capable of initiating the NRR active centers, and charge accumulation on the nanostructured diamond surface enables further enhancement of catalytic activity through reducing the reaction free energy for the rate‐determining step of the NRR on certain surfaces.^[^ ^53^ ^]^

#### 1.2.4 Organic Electrosynthesis

The chemical stability and low background currents that make diamond useful in aqueous environments extend more broadly into nonaqueous media and enable new applications such as electrosynthesis of organic compounds in a sustainable manner. Some of the earlier electrochemistry results have been summarized previously.^[^ ^54^ ^]^ Since then, processes with even better Faradaic efficiencies and high selectivity have been reported, such as late‐stage arene alkenylations^[^ ^55^ ^]^ and the synthesis of hydroxyl quinazolin‐4‐ones.^[^ ^56^ ^]^ A unique reaction observed on diamond is the electrochemical methoxylation, which is due to the formation of alkoxyl radicals at its surface.^[^ ^57^, ^58^ ^]^ Based on diamond's semiconducting nature, the electrochemical properties of diamond electrodes under oxidizing and reducing conditions are also dependent on the doping and the type of the charge carriers (negative electrons or positive holes) within the bulk.^[^ ^59^ ^]^

#### 1.2.5 Electrochemical Energy Storage

Another area where the electrochemical stability of diamond plays a key role is in electrochemical energy storage systems such as batteries and supercapacitors (SCs). Batteries store energy through changes in oxidation/reduction reactions, while SCs store energy by changing the spatial organization of ions at the electrode surface. While diamond's tightly packed lattice limits the interaction of Li ions, conductive diamond, especially when nanostructured, can provide a protective coating and can be used as a high‐surface area electrode.^[^ ^60^ ^]^ Consequently, both batteries and supercapacitors can benefit from diamond's electrochemical stability and ability to be made electrically conductive by doping with boron, nitrogen, or phosphorus during growth. Diamond films, diamond composites, and diamond nanostructures, including nanoparticles, wires, foams, and fibers, have been applied as electrode materials. A unique double‐layer nanodiamond thin film has been designed as interfacial protection for Li metal anodes, ensuring uniform ion flux and protection against formation of dendrites, a major safety problem when recharging lithium‐ion and lithium‐metal batteries.^[^ ^61^, ^62^ ^]^ An N‐doped ultrananocrystalline diamond anode coated with a natural graphite (NG)‐Cu composite inhibited the co‐intercalation of the electrolyte into NG (a major problem with battery stability), while its capacity retention was increased.^[^ ^61^, ^63^ ^]^ The introduction of detonation nanodiamond particles into a typical carbonaceous anode matrix^[^ ^64^ ^]^ and multiwalled carbon nanotubes/LiCoO_2_,^[^ ^65^ ^]^ as well as into the electrolyte to adsorb Li‐ions^[^ ^66^ ^]^ improved the performance of the constructed batteries, such as Li storage capacity, cycling performance, and stability of the Li‐ion batteries.^[^ ^67^ ^]^ In addition to Li‐ion batteries, diamond has been applied to fabricate Zn‐air batteries^[^ ^68^ ^]^ and nuclear batteries.^[^ ^69^, ^70^ ^]^

**Figure 2:**
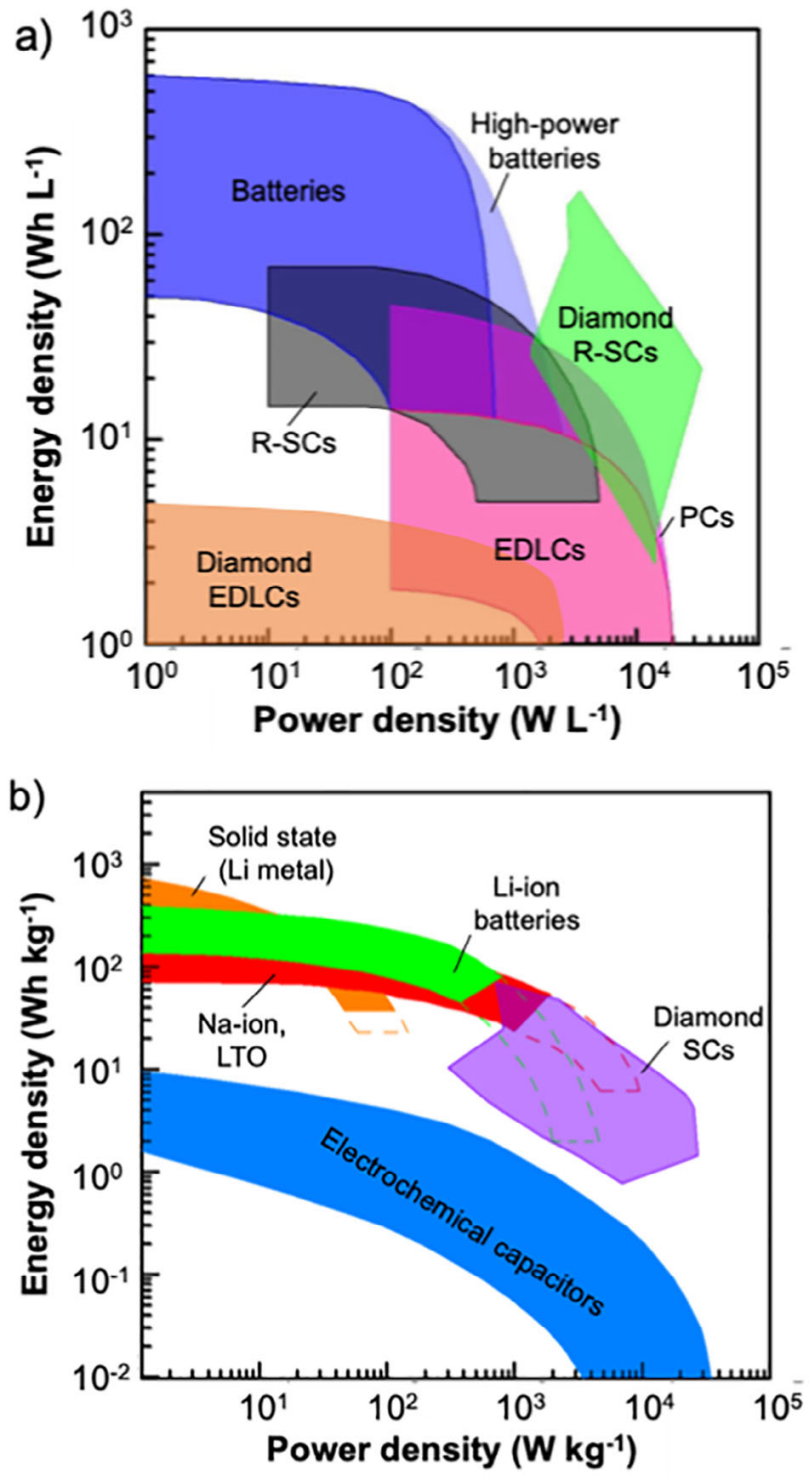
Comparison of energy densities and power densities of diamond supercapacitors (SCs), diamond electrical double‐layer capacitors (EDLC), pseudocapacitors (PCs), and redox electrolytes enhanced SCs (R‐SCs) with those of other energy storage devices when the volumes a) and masses b) of diamond electrodes are applied for these calculations.^[^ ^27^ ^]^ Adapted with permission from Ref. [27]. Copyright 2022, American Chemical Society.

Among the major classes of electrical storage devices, batteries have high energy densities but suffer from low power densities, while SCs have high power densities but lower energy densities. Nanostructured diamond electrodes provide an opportunity to achieve both high energy density and high power density by coupling high‐surface‐area nanostructured diamond electrodes with soluble redox‐active electrolytes to form redox electrolyte‐enhanced SCs (R‐SCs).^[^ ^71^, ^72^, ^73^ ^]^ Diamond‐containing electrochemical energy devices such as electrical double layer capacitors (EDLCs), pseudocapacitors (PCs), and R‐SCs show superior performance for a range of performance metrics, including their capacitances, lifetime or cycling stability, gravimetric power densities (*P*_g_), volumetric power densities (*P*_v_), gravimetric energy densities (*E*_g_), and volumetric energy densities (*E*_v_).^[^ ^27^, ^74^, ^75^ ^]^ As expected, diamond SCs are highly stable, as evidenced by the fact that the capacitances of most diamond SCs remain unchanged even after 10 000 charging/discharging cycles. These energy‐ and power‐density values were further integrated into existing gravimetric and volumetric Ragone plots,^[^ ^75^ ^]^ where the variation of powder densities of different electrochemical energy storage devices (e.g., supercapacitors and batteries) are plotted as function of their energy densities. Importantly, diamond SCs also exhibited high power and energy densities when the volumes (Figure 2a) and masses (Figure 2b) of these diamond electrodes were taken into consideration. Especially, the *E*_g_ and *E*_v_ values of diamond R‐SCs are higher than those of most SCs, while their *P*_g_ and *P*_v_ values are bigger than those of batteries.^[^ ^27^, ^74^, ^75^ ^]^ Diamond R‐SCs were then also named as battery‐like SCs or supercapbatteries, and the high charge storage capacity was explained by fast Faradaic reactions of redox species occurring at the diamond surface or confined redox species inside diamond electrodes. Further matching of redox electrolytes (e.g., type or amount) with diamond electrodes (e.g., size and density of pores/channels) is expected to construct diamond supercapacitors with even higher performance.^[^ ^27^, ^74^, ^75^ ^]^

#### 1.2.6 Diamond for Quantum Science

**Figure 3:**
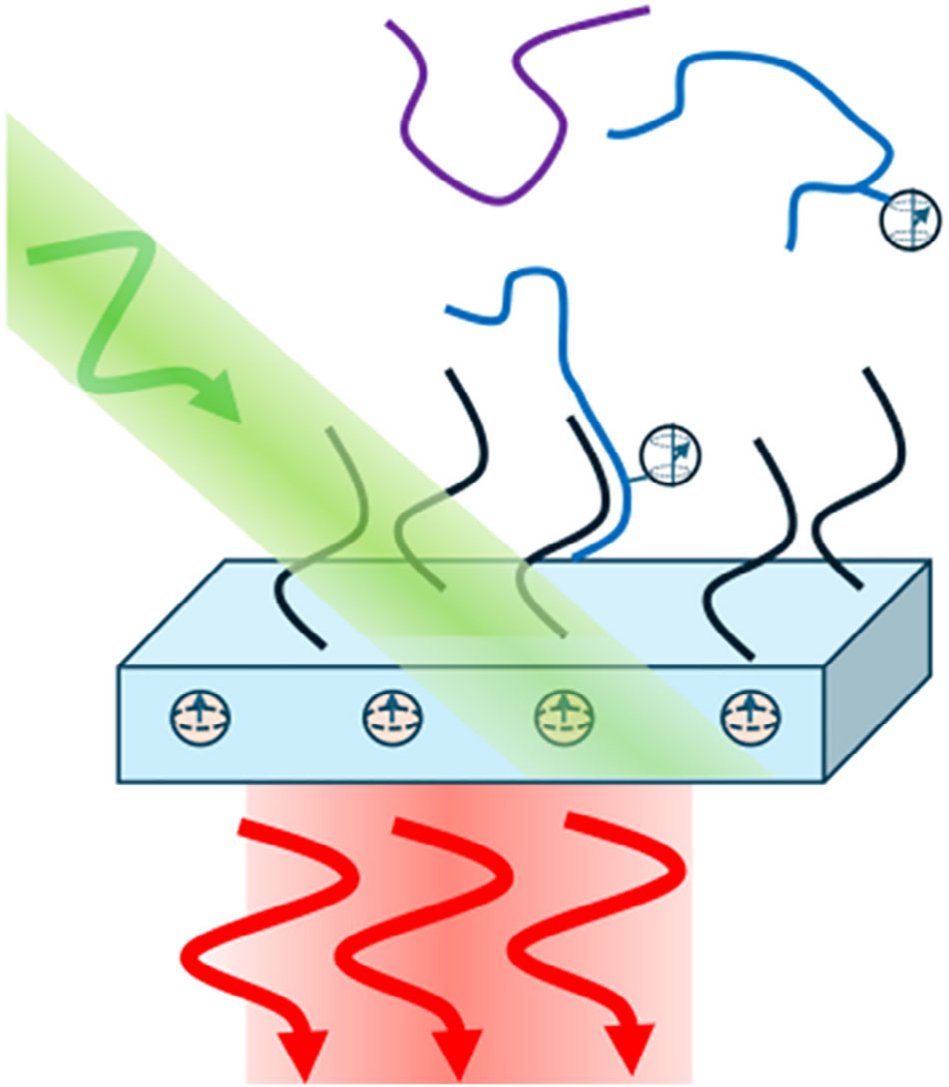
One approach to using NV centers for chemical sensing. Diamond surfaces and nanoparticles with near‐surface NV centers can be functionalized to expose DNA or other biomolecules (black). If complementary molecules (blue) labeled with spin centers bind to the surface, the NV centers in the vicinity are affected. The NV spin properties can be prepared and read out using fluorescence.

While defects in solid‐state materials are usually considered deleterious, several defects in diamond have remarkable properties that can serve as a basis for new applications. Among these, the nitrogen vacancy (NV) center has attracted the most attention.^[^ ^76^, ^77^ ^]^ The NV center consists of a substitutional N atom with an adjacent vacancy that can be in a negatively charged (NV^−^) or neutral state (NV^0^). The NV^0^ and NV^−^ centers, along with the related silicon vacancy (SiV) center, can yield very high photoluminescence quantum efficiencies and exhibit remarkable chemical, thermal, and photo‐stability^[^ ^78^, ^79^, ^80^ ^]^ making them ideal optical tags for a wide range of applications. The NV and SiV centers have attracted a great deal of attention because the electrons are paired into a triplet ground state, but the spin state can be easily manipulated optically using even weak, mid‐visible optical fields or by weak magnetic fields, allowing preparation of NV centers with nearly 100% spin polarization.^[^ ^76^, ^81^ ^]^ Furthermore, the very high sensitivity of optical (fluorescence) and/or electronic detection allows the preparation and characterization down to the single‐spin level.^[^ ^82^, ^83^ ^]^ Many of the same methods used to probe electron spins in electron spin resonance and nuclear magnetic resonance can be applied to these NV centers, but with dramatically improved sensitivity approaching the single‐spin limit.^[^ ^83^, ^84^, ^85^ ^]^ Several other color centers have also been explored, such as the silicon‐vacancy (SiV) center.^[^ ^77^, ^86^ ^]^ A common feature of these centers is that unpaired spins in the diamond lattice can have long spin coherence times making them attractive for chemical sensing with very high resolution.^[^ ^87^, ^88^, ^89^ ^]^ Furthermore, in many cases it is possible to both prepare specific spin states and also to probe the spin state using optical methods, allowing exquisitely sensitive detection of spin properties down to the single‐spin level. Figure 3 shows one very simplified implementation using spin‐tagged molecules, combined with surface functionalization with a molecular recognition agent (black) to provide chemical selectivity.

While NV^−^ and related color centers are extraordinarily sensitive probes of magnetic fields, coupling between proximal spins is of interest for applications such as quantum computing. Moreover, the interaction of NV centers with spins on nearby molecules can serve as a basis for new types of spin‐based quantum sensors with myriad applications in chemistry, physics, and emerging quantum technologies.^[^ ^90^, ^91^, ^92^, ^93^ ^]^ For example, a nonzero electronic or nuclear spin on chemical species in an aqueous solution can alter the coherence properties of NV centers located close to the diamond–water interface.^[^ ^78^, ^94^, ^95^ ^]^ In this scenario, the coherence properties of the NV centers serve as an optical readout to reveal information about the presence of spins in the aqueous‐phase chemical environment. Recent measurements of the T_1_ relaxation time of NV^−^centers in ∼100 nm fluorescent nanodiamonds showed a decrease in the T_1_ relaxation time in the presence of reactive oxygen species and other radicals.^[^ ^96^, ^97^ ^]^

#### 1.2.7 Diamond in Biology and Medicine

Diamond finds widespread utility in biology and biomedicine. Suitably purified and functionalized diamond materials show little to no toxicity and exhibit very good biocompatibility.^[^ ^98^, ^99^ ^]^ Furthermore, diamond nanoparticles and films can be covalently functionalized with organic and/or biological molecules, providing tunable molecular recognition properties. Finally, the introduction of fluorescence chromophores either through external functionalization or through incorporation of color centers such as the NV^−^ center, provides the ability to track the location of diamond nanoparticles for various types of bio‐imaging studies. These properties have led to a variety of applications in drug delivery, tissue engineering, bioimaging, and sensing (see below). The use of light‐emitting color centers in diamond for bioimaging leads to nonbleachable biolabels that are embedded in the material matrix, thus enabling an unaltered surface of the material and preventing cleavage of the label.^[^ ^100^ ^]^ The accessibility of the diamond surface for chemisorbed or covalently bound functional moieties allows the formation of hybrid materials that combine the properties of diamond with the functions of these units, such as antibodies,^[^ ^101^, ^102^ ^]^ growth factors,^[^ ^103^ ^]^ drugs,^[^ ^104^ ^]^ or spin labels.^[^ ^105^, ^106^, ^107^ ^]^ Imaging has been achieved in a variety of environments, such as in vitro, in cells or in animals. It was found that NDs can cross the blood‐brain barrier,^[^ ^108^ ^]^ and cell autofluorescence can be reduced using NDs as labels.^[^ ^109^ ^]^ Imaging can be done over extended periods of time due to the nonbleaching behavior of color centers in nanodiamonds.^[^ ^110^ ^]^ The NV^−^ centers in diamond, as described above, can be used as highly stable chromophores in bulk and nanodiamond. The unique spin properties of these centers allow them to be used as quantum sensors^[^ ^111^, ^112^, ^113^ ^]^ for various magnetic‐resonance studies with sensitivity approaching the single‐spin level. The ability to track nanodiamonds optically can be combined with drug delivery to monitor the fate of delivery vehicles inside the cells.^[^ ^114^ ^]^

Drug delivery in general has been a focus for application of nanodiamonds. The loading of different, typically water‐insoluble, drugs onto nanodiamonds enables their efficient uptake and release. Such systems have been used to deliver, e.g., antitumor drugs such as doxorubicin, epirubicin, and paclitaxel.^[^ ^115^, ^116^, ^117^ ^]^ For some of these systems, large animal studies have already shown the efficacy and safety of the conjugates.^[^ ^118^ ^]^ Besides delivery of drugs, the use of diamond surfaces for tissue engineering is another important aspect. Examples include the use in bone tissue engineering for the differentiation of stem cells and the growth of new bone in large defects,^[^ ^119^, ^120^ ^]^ or the formation of networks of neurons.^[^ ^121^, ^122^ ^]^

## 2 Fundamentals of Diamond Chemistry

The properties of diamond materials are strongly related to the chemistry of the material. Starting from the processes occurring during the growth of diamond to the effects on agglomeration and biocompatibility, the surface chemistry dictates the behavior of the material. Being able to control the chemistry of diamond ensures the control of its unique properties, such as electron affinity, the quality and concentration of doping, and the interaction with its environment, biological or not.

### 2.1 Chemistry of Diamond Growth

Bulk diamond materials are typically grown either under conditions of high temperature and high pressure (“HPHT diamond”) or via chemical vapor deposition (CVD). Significant advances in both these methods have greatly expanded the range of diamond materials that are accessible for research and applications and have greatly reduced their cost. In addition, very small diamond nanoparticles have emerged as a novel form of diamond referred to as “detonation nanodiamond” or “DND.” DND is formed by the detonation of explosives in confined environments, transiently reaching the thermodynamic stability region of diamond. Here we briefly summarize some of the fundamentals of CVD diamond growth and of the diamond surface chemistry that are the foundations for diamond's many applications.

**Figure 4:**
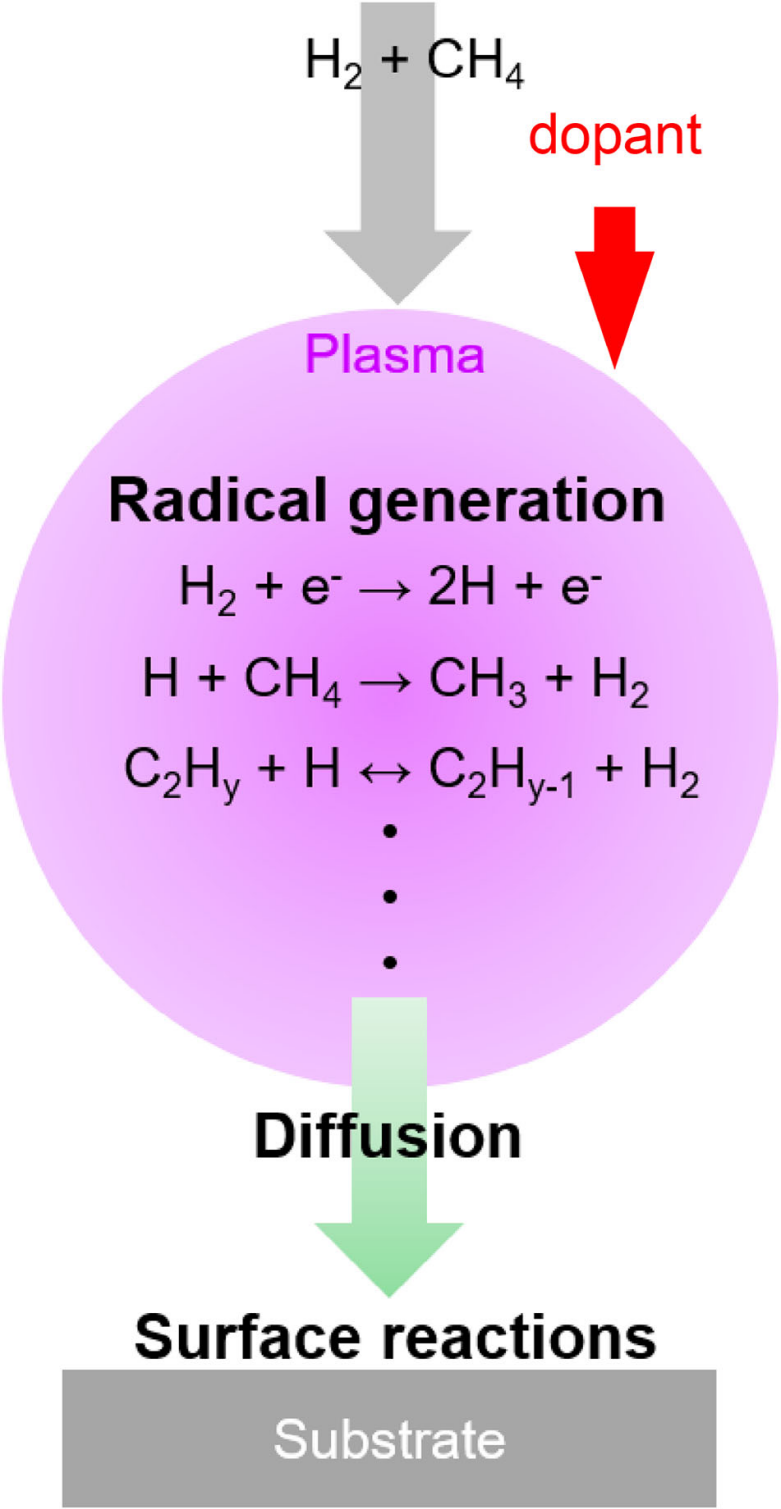
Illustration of chemical processes of chemical vapor deposition of diamond on substrates.^[^ ^123^, ^124^ ^]^ Adapted with permission from Refs. [123, 124]. Copyright 2019, Springer Nature Switzerland AG.

CVD represents a widely employed and inexpensive technique to grow diamond thin films and, more recently, larger bulk samples with different crystallinities and sizes. It is particularly important for chemistry‐related applications because CVD diamond can be grown as thin films on a range of conductive substrates (e.g., metals, silicon). CVD growth is carried out under highly nonequilibrium conditions under which diamond growth is favored kinetically over growth of graphite (Figure 4). CVD is based on the decomposition of methane (CH_4_) or other carbon‐containing precursors (CO, CO_2_, C_2_H_5_OH, graphite) with the aid of other energy sources, typically in a large excess of H_2_ gas. High temperatures and/or microwave or radio‐frequency plasmas are used to dissociate the precursor molecules into atomic hydrogen and various radical species (Figure 4). Under common growth conditions using CH_4_ + H_2_ as growth precursors, H atoms formed from dissociation of H_2_ in the plasma initiate hydrogen abstraction reactions that produce gas‐phase activated methyl radicals (CH*_x_*, *x* = 0, 1, 2, 3), which combine to form activated C_2_H*_y_* (*y* = 0, 1, 2, 3, 4, 5, and 6) species in the gas phase. Both CH*_x_* and C_2_H*_y_* radicals are regarded as precursors for diamond growth. These reactive species then react on the surface to produce a diamond film. The predominant role of radical chemistry differs from other types of CVD processes frequently used in semiconductor processing. A key aspect of diamond growth involves having the diamond surface nearly fully terminated by hydrogen. Hydrogen abstraction at the diamond surface leaves a small number of under‐coordinated carbon atoms as active sites. When these radicals diffuse to a diamond substrate surface or a substrate (e.g., Si) covered with diamond seeds, a complex series of reactions happens, sp^3^ diamond is then formed preferentially, leading to the growth of diamond films.

A key aspect of diamond growth is the presence of a high concentration of hydrogen radicals (atomic hydrogen) in the gas phase. H atoms are typically generated by thermal dissociation on a hot filament of W or Ta (“hot‐filament CVD”) or in plasma by electron impact, collisional energy transfer, etc. (“plasma‐enhanced CVD”). In plasma, the external energy input couples to free electrons, which dissociate and ionize the input reactant gases into highly reactive growth precursors. Hydrogen radicals play a role in the growth of high‐quality diamond films by removing nondiamond, such as graphite, formed on the diamond surface. As such, diamond growth and selectivity for diamond over graphite depend both on the deposition of growth precursors and on simultaneous etching reactions. Diamond is etched by reactions with hydrogen radicals, although the etching rate is lower than that of nondiamond carbon. Consequently, CVD diamond growth is accompanied by the reactions of hydrogen abstraction and adsorption and by etching on diamond surfaces, which limits chemisorption of diamond precursors and diamond nucleation. The composition and the concentration of gaseous precursors are the crucial factors to determine the features of as‐grown diamond films (e.g., composition, surface morphology, and microstructure).^[^ ^123^, ^124^ ^]^

Many of the practical applications of diamond take advantage of its ability to be doped into a conductive state. As a wide band gap material, pure diamond is highly insulating. By doping it with heteroatoms that donate electrons (such as P or N, referred to as “donors”) or withdraw electrons (such as B, referred to as an “acceptor”), diamond can be made conductive. Many applications of diamond require precise control of the carrier type (electrons vs. holes) and the conductivity and concentration to control the electrical resistivity of diamond semiconductors for the realization of diamond‐based electronic devices. Diamond surfaces can also be made conductive through a process known as surface‐transfer doping (described below), while diamond thin films are frequently conductive due to the presence of graphitic sp^2^‐hybridized carbon in the film. However, highly controlled electronic properties can only be achieved through controlled introduction of dopants (“impurities”) such as B, N, and/or P.^[^ ^125^, ^126^ ^]^

Doping of diamond films is relatively inefficient both because of poor incorporation of dopant atoms into the diamond lattice in the proper site and because only a small fraction of dopant atoms in the current site are in the required charge state. The energies associated with dopant activation for P, N, and B are 1.7, 0.57, and 0.37 eV, which are large compared with room‐temperature thermal energies (0.026 eV at 300 K).^[^ ^127^ ^]^ Doping with phosphorus leads to n‐type diamond with high resistivity, while doping with boron results in the growth of a p‐type semiconductor. Boron and phosphorus doping is typically carried out by introducing diborane (alternatively, trimethylboron or trimethylborate) and phosphine (or tris‐*tert*‐butyl phosphine) gases, respectively. Carrier mobility increases with increasing boron or phosphorus concentrations in diamond films, reducing the resistivity of diamond. The BDD films can be doped in a way yielding semiconducting properties; at higher boron concentrations, metallic properties can be achieved (particularly useful for electrochemistry), and even, at low temperatures, it becomes superconducting.^[^ ^128^ ^]^ However, it is still challenging to dope diamond with other dopants at useful concentrations, often due to the large atomic size of the dopant atoms.^[^ ^129^ ^]^ It must be mentioned here that although thin polycrystalline diamond films are sufficient for electrochemistry, one of the main challenges is to produce large single‐crystal diamond wafers of high purity for semiconductor applications.

While much has been learned about the chemistry of diamond growth and doping, many aspects of the underlying mechanisms by means of CVD are still not well understood. To elucidate such a mechanism, both vapor‐phase and surface reactions need to be understood. Observation of the growth surface is crucial for elucidation of the growth mechanism because the growth process influences the structure of the growth surface. Evaluations based on optical emission spectroscopy and mass spectrometry (MS) have to be conducted on the production and diffusion processes of hydrogen, CH*_x_* radicals, and C_2_H*_y_* radicals. Although recent simulation results of the distribution of radical, gas, and electron temperatures in plasmas provide some information on vapor‐phase reactions in the CVD diamond process, microscopic experimental results are still needed to reveal how those radicals arrive at diamond surfaces, migrate, and react with hydrogen, terminating the surface and/or carbon. Unfortunately, it is extremely challenging to identify the involved processes because of the difficulty of conducting in situ characterizations in plasma environments. Scanning probe microscopy (SPM), low‐energy electron diffraction (LEED), Fourier transform‐infrared spectroscopy (FT‐IR), and electron energy loss spectroscopy (EELS) provide physical and chemical information on surfaces at the atomic level and are powerful tools for the study of diamond CVD growth.^[^ ^123^, ^124^, ^129^ ^]^

In terms of CVD reactors, hot‐filament CVD has been applied to large‐scale industrial processes because of its simple system configuration and ability to coat large areas and complex shapes. However, hot‐filament CVD growth of diamond films must be carried out at lower gas temperatures and pressures than those of plasma CVD because of the upper temperature limit of the filament materials and the low production rate of hydrogen radicals. This leads to relatively low growth rates of diamond films compared to diamond growth by plasma‐enhanced CVD (PECVD). Using PECVD, comparably fast homoepitaxial diamond growth has been realized. Additionally, both p‐ and n‐type diamond films have been reproducibly grown by means of PECVD.^[^ ^129^ ^]^

### 2.2 Surface Chemistry of Diamond

**Figure 5:**
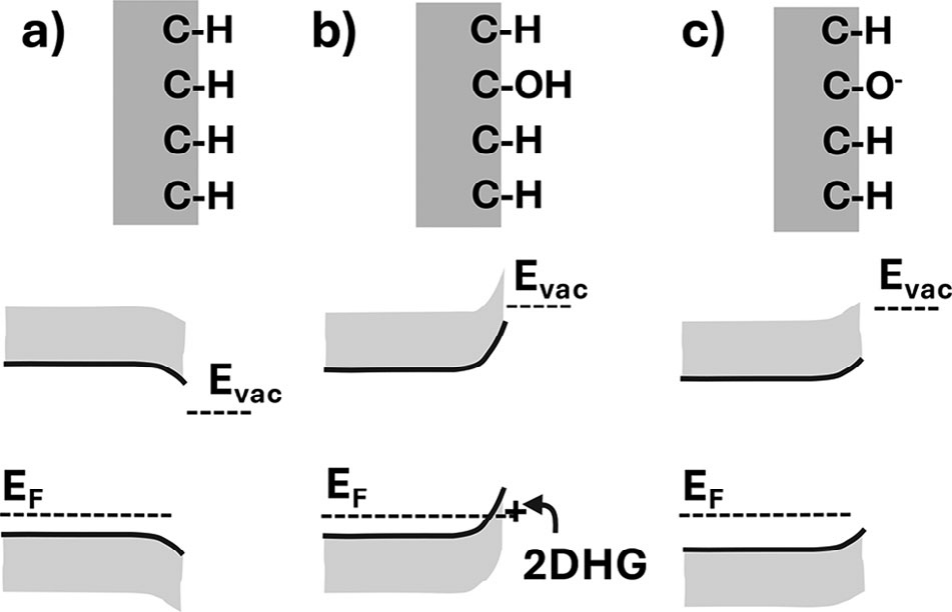
Influence of surface termination on diamond energy bands and vacuum level: a) As‐grown, H‐terminated diamond; b) H‐terminated diamond after air exposure with formation of 2D hole gas (2DHG); and c) wet‐oxidized diamond with deprotonated surface ─OH groups inducing upward band‐bending.

Many of diamond's applications are based on its surface properties, which can be widely tuned with recent advances in diamond surface chemistry. Fractured or cleaved diamond surfaces expose a very high density of under‐coordinated surface atoms. Cleaving diamond to expose (111) surfaces, for example, produces under‐coordinated C atoms that are separated by only 0.252 nm, smaller than the effective size of most molecules. The exposed atoms are very reactive, but because of the very small lattice spacing, perfect surface termination can only be achieved with species that have a small effective footprint. Termination of the surface with a layer of atomic hydrogen is by far the most widely studied surface termination (Figure 5).^[^ ^130^ ^]^ In addition to hydrogen's small atomic size, the growth conditions for diamond epitaxial and polycrystalline films typically present a large excess of atomic hydrogen, such that “as‐grown” diamonds are typically hydrogen terminated. Consequently, hydrogen‐terminated diamond is most frequently used as a starting point for further chemical functionalization with molecular species.

The chemical nature of diamond surfaces has a great impact on diamond's near‐surface electrical properties that are key to several of diamond's important technological applications. As depicted (Figure 5), these impacts can be divided into two spatial regimes: the space‐charge layer extending tens to hundreds of nanometers under the surface and the surface dipole associated with the atoms at the last atomic layer at the solid‐vacuum interface. Charge exchange between diamond and adjacent surfaces or bulk materials (e.g., water, solids) due to differences in electronegativity or electrochemical potential leads to a net charge (“space‐charge”) in the subsurface region and controls the location of diamond's band edges with respect to the Fermi level, commonly referred to as “band‐bending.” Band bending impacts the conductivity and can alter the charge state of species in the near‐surface region. The local chemical interaction of the surface C atoms with H, F, O, or other surface atoms also leads to a highly localized surface dipole; this dipole, largely confined to the very outermost chemical bonds, impacts processes such as electron emission and the energy of the vacuum level (the energy of an electron just outside of the solid).^[^ ^131^ ^]^ Notably, H‐terminated diamond has a vacuum level below the conduction band; this unique property of negative electron affinity is of especially great importance for photo‐stimulated or thermally stimulated electron emission processes.^[^ ^130^ ^]^

The ability to control diamond's electrical properties through changes in surface chemistry has attracted a great deal of attention, ranging from electronics to sensing.^[^ ^132^, ^133^, ^134^, ^135^ ^]^ When H‐terminated diamond surfaces equilibrate with air or thin water layers, charge exchange leads to a very thin, positively charged (p‐type), highly conductive surface layer in which the concentration of holes (positive charges) is so large that it is frequently described as a 2D hole gas.^[^ ^136^, ^137^, ^138^ ^]^ Similarly, H‐terminated nanoparticles in water are stabilized by charge exchange and protonation of surface sp^2^‐(graphitic) carbon, leaving them with a positive charge, helping to stabilize them in colloidal suspensions.^[^ ^139^ ^]^ Adsorption of NO_2_ gas leads to the formation of surface NO_3_^−^ ions and even more robust upward band bending.^[^ ^136^ ^]^ The intentional chemical manipulation of the near‐surface conductivity has applications ranging from electronics to chemical sensing.^[^ ^140^ ^]^ For example, if a probe molecule (e.g., single‐stranded DNA) is covalently linked to a diamond surface, hybridization with a complementary DNA strand leads to a change in the conductivity of the underlying diamond.^[^ ^134^ ^]^ This concept can be the basis for chemical sensors that directly transduce chemical information into electronic signals.^[^ ^3^, ^140^, ^141^ ^]^

Band‐bending associated with surface termination also impacts applications in chemical sensing by modifying the charge state of species at or near the surface. Recent interest in the use of nitrogen‐vacancy centers as luminescent centers or quantum spin probes has driven especially great interest in controlling the chemistry of the surface and near‐surface space‐charge region. While hydrogen atoms can yield almost perfect terminations of the diamond surface, the resulting band‐bending can cause the transformation of NV centers into the NV° charge state instead of the desired NV^−^ charge state. This has driven intense interest in different approaches to making diamond surfaces with alternative surface terminations. Fluorine‐terminated surfaces are of interest because the small size and high C─F bond strength are predicted to yield surfaces with good chemical stability and a large surface dipole‐oriented opposite to that of the H‐terminated surface.^[^ ^131^ ^]^ The recent emergence of diamond as a material for quantum studies has led to increased interest in controlling the surface termination and, in particular, surfaces with Li,^[^ ^142^ ^]^ N, O,^[^ ^143^, ^144^ ^]^ or ─OH termination. Diamond surfaces terminated with amino groups (─NH_2_) have been made using an ammonia plasma^[^ ^145^ ^]^ at the surface for electron emission from the conduction band. Making high‐quality oxygen‐terminated diamond is challenging because oxygen's bivalent coordination and larger size lead to multiple surface species, including ether linkages, carbonyl groups, and alcohol (─OH) surface groups.^[^ ^142^, ^143^, ^144^ ^]^

**Figure 6:**
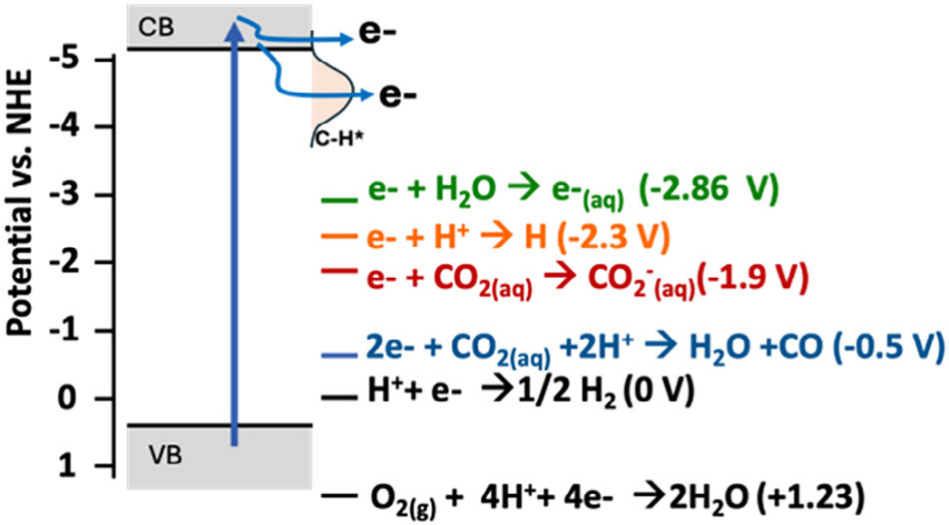
Electrochemical reduction due to electron emission into water. Electron emission occurs from the conduction band and through a surface state associated with the C–H surface termination. Electrons in water form species such as the solvated electron (e^−^_(aq)_), a highly potent reducing agent that can induce many energetically demanding reactions.^[^ ^41^ ^]^ The term of NHE refers to a normal hydrogen electrode. Adapted with permission from Ref. [41]. Copyright 2013, Springer Nature Limited.

The surface chemistry of diamond also has a large impact on diamond's electron emission properties. One of the unique properties of diamond is that H‐terminated diamond has negative electron affinity. When terminated with hydrogen atoms, the electronegativity difference between C and H leads to a C^−^─H^+^ surface dipole. While the vast majority of solid‐vacuum interfaces have increased electron density at the surface that creates a barrier to electron emission,^[^ ^146^ ^]^ for H‐terminated diamond, the positive charge on the surface H atoms allows electrons to more easily escape from the solid into the vacuum. When combined with downward band‐bending, this leads to a diamond‐vacuum interface in which there is no barrier for electron emission.^[^ ^138^ ^]^ As depicted in Figure 6, in this situation electrons excited into the conduction band by heat, light, or other excitation sources can be emitted in a barrier‐free manner into vacuum, either directly or mediated by surface states such as the C─H antibonding state associated with H‐termination of diamond.^[^ ^138^, ^147^ ^]^ Recent studies have extended this into other types of ambient environments, including gases and even into water, forming solvated electrons (Figure 6).^[^ ^41^, ^148^, ^149^ ^]^ As depicted in Figure 6, diamond's energy bands can be placed on the standard electrochemical potential scale. Here it can be seen that electrons emitted from diamond's conduction band have a very large negative potential. Once in water, reorganization of the surrounding water molecules rapidly forms solvated electrons e^−^_(aq)._ Solvated electrons are a highly potent reducing agent with a reduction potential of −2.86 V, sufficient to induce many difficult reduction reactions such as the reduction of H^+^ to the hydrogen atom H• (a critical intermediate in ammonia synthesis) and reduction of CO_2_.^[^ ^150^ ^]^

Solvated electrons produced from diamond single crystals, polycrystalline diamond thin films, and suspensions of diamond nanoparticles in water have been shown to synthesize NH_3_ from N_2_ and to reduce CO_2_ with high efficiency.^[^ ^39^, ^40^, ^41^, ^148^, ^151^, ^152^, ^153^ ^]^ Solvated electrons in water produced by photoemission from diamond can be directly detected spectroscopically using transient absorption methods, directly confirming their presence.^[^ ^149^, ^154^ ^]^

**Figure 7:**
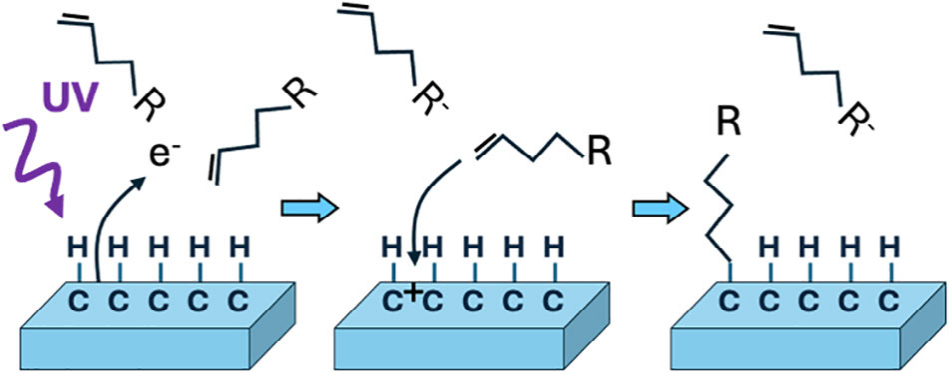
Photochemical grafting of terminal alkene to H‐terminated diamond surface initiated by ultraviolet light. Adapted with permission from Ref. [130]. Copyright 2005, American Chemical Society.

The modification of diamond with different molecular species plays a critical role in many of diamond's applications in chemistry and biology. Unlike the vast majority of molecule‐semiconductor interfaces, *C*_diamond_‐*C*_molecule_ bonds formed at the interface between diamond surfaces and organic molecules are intrinsically stable in water, highly resistant to any type of degradation process even under the most extreme conditions of salt, pH, or redox potential. One common approach to forming these stable interfaces is through the reaction of organic molecules bearing terminal vinyl groups (i.e., R─CH═CH_2_) reacting with hydrogen‐terminated diamond surfaces.^[^ ^1^ ^]^ Detailed mechanistic studies showed that this grafting reaction can be readily initiated using ultraviolet (UV) light, even somewhat below the bandgap, which induces diamond to emit electrons into the adjacent reactant medium (Figure 7).^[^ ^155^ ^]^ This leaves the diamond surface with carbocations that are reactive toward nucleophilic groups such as the terminal vinyl groups. The reactions on diamond are self‐terminating at the one‐monolayer level and occur at rates that are dependent on the electron affinity of the molecules in the adjacent liquid medium. The UV‐initiated grafting approach has been demonstrated to make a wide range of functional interfaces that can then be subjected to further chemical reactions to link DNA,^[^ ^1^, ^156^ ^]^ proteins,^[^ ^157^ ^]^ electro‐active catalyst molecules,^[^ ^158^, ^159^, ^160^ ^]^ and molecular spin probes^[^ ^161^ ^]^ to diamond surfaces.

### 2.3 Surface Chemistry of Diamond Nanoparticles

Much of the surface chemistry of diamond is intrinsic to sp^3^‐hybridized carbon and therefore can frequently be applied across different crystal faces and to single‐crystal, polycrystalline, and nanodiamond.^[^ ^156^, ^162^ ^]^ However, there are differences in reactivity and practical applications of diamond nanoparticles of various sizes.^[^ ^163^ ^]^ Larger (>10 nm) diamond nanoparticles are typically made by milling of larger diamonds followed by screening (e.g., centrifugation) to separate them into specific size ranges. These nanodiamonds expose multiple crystal planes and frequently resemble glass shards in overall appearance. They have high crystallographic quality, although there are some size‐dependent properties due to the possible presence of sp^2^‐hybridized carbon at their surfaces. Very small nanoparticles of ∼4–5 nm diameter are also widely used; these diamond nanoparticles are typically prepared by detonating explosives in a closed vessel, leading to high‐pressure shock waves that produce diamond nanoparticles.^[^ ^164^ ^]^ These nanoparticles are frequently referred to as “detonation nanodiamond” (DND) and are of great interest for application as ultra‐stable bioprobes. The presence of multiple surface crystallographic sites, high nitrogen content (from the nitrogen‐containing explosives used), and frequent presence of sp^2^‐hybridized surface carbon cause detonation diamond nanoparticles to have a surface chemistry that is somewhat different from that of other forms of diamond. The surface sites can be more readily oxidized, leading to carbonyl and alcohol groups that can be used as starting points for further functionalization.^[^ ^162^ ^]^ These features render detonation nanodiamond a class of its own with regard to chemical properties. Its reactivity and its interactions with physiological systems differ significantly from bulk diamond and nanoparticles of other origin.

The surface chemistry of diamond nanoparticles and related materials has seen tremendous development over recent years. Setting out from harsh oxidative treatments and ill‐defined functionalization protocols, current achievements lead to highly defined and multifunctional nanodiamond particles for a multitude of applications as above.

The efforts of earlier research have been extensively reviewed.^[^ ^165^, ^166^ ^]^ It was shown that nanodiamonds can be functionalized using not only surface groups for the grafting of functional moieties but also direct grafting onto the carbon surface, e.g., by replacing hydrogen atoms or applying cycloaddition chemistry on surface‐reconstructed particles, are viable approaches for direct C─C coupling.^[^ ^167^, ^168^, ^169^ ^]^ The stability of directly grafted moieties leads to superior performance, e.g., in biomedical applications.^[^ ^170^, ^171^ ^]^ Recently, the tools and methods for nanodiamond functionalization became even more tailored using mild and biocompatible strategies as well as approaches to multifunctional materials.^[^ ^166^, ^172^, ^173^ ^]^

In contrast to the other carbon nanomaterials such as fullerenes, nanotubes, and graphene, functionalization of the diamond surface does not lead to a change in hybridization of the carbon surface atoms and thus alters the electronic properties of the material much less. However, functionalization still influences the position of the valence and conduction band edges (band‐bending) and the electron affinity of the surface.^[^ ^174^, ^175^, ^176^ ^]^

When comparing the surface chemistry of flat diamond surfaces, such as bulk or thin‐film material, with that of diamond nanoparticles, a clear difference in chemical behavior is observed.^[^ ^177^, ^178^ ^]^ Nanoparticles have higher concentrations of under‐coordinated sites and reduced steric crowding compared to flat surfaces. Consequently, while many reactions on flat surfaces require rather harsh conditions, the reactivity of nanoparticles is significantly higher, such that milder processes can be employed. Additionally, surface loadings tend to be higher not only due to the increased specific surface area but also related to that very reactivity.^[^ ^179^ ^]^ The higher reactivity of nanodiamond is largely driven by the fact that the surface free energy of diamond nanoparticles decreases with decreasing diameter, independent of the actual shape of the crystal.^[^ ^180^ ^]^ Several theoretical investigations have covered the different reactivity of the different surface facets, which still play a role even for small nanoparticles. While their actual size is very small, they govern the surface charges, the interaction with different reactants, as well as the theoretically achievable loadings.^[^ ^181^, ^182^, ^183^ ^]^

It was found that the^[^ ^180^ ^]^ facet exhibits different chemical affinity for certain reactants than the^[^ ^158^, ^159^, ^160^, ^161^ ^]^ facet with a denser packing of surface groups, depending on the reactant.^[^ ^182^ ^]^

Recently, the initial termination of the diamond surface was recognized as one of the important factors for the electron emission properties as well as the charge states of color centers close to the surface of diamond. In short, it can be said that aminated and fluorinated surfaces promote both the negative electron affinity of the surface and the stabilization of the negative charge state of NV centers.^[^ ^184^, ^185^ ^]^

Several methods for a more homogeneous termination of diamond have been proposed. It was shown that nitrogen‐containing groups, i.e., amino and imino groups have a clear influence on the electronic properties of the surface.^[^ ^145^, ^186^, ^187^ ^]^ Partial fluorination was achieved, e.g., by using SF_6_ plasma,^[^ ^188^ ^]^ and photofluorination.^[^ ^189^ ^]^ Oxygen termination was originally achieved through thermal oxidation in air or plasma oxidation. Milder conditions in liquid phase enable the homogeneous establishment of different types of oxygen‐containing groups, such as OH and COOH.^[^ ^190^, ^191^, ^192^ ^]^

**Figure 8:**
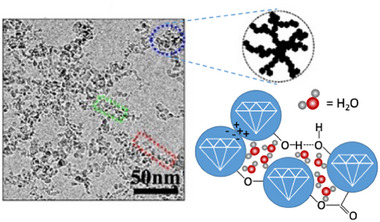
The surface termination of diamond nanoparticles also plays an important role in the aggregation behavior, e.g., in aqueous solution. Left: TEM image of detonation nanodiamond agglomerates. Right: structure of the agglomerates and the underlying chemical interactions. TEM image taken with permission from Ref. [198]. Copyright 2009, Royal Society of Chemistry.

The surface termination also plays a crucial role in the formation of the different types of aggregates and agglomerates of diamond nanoparticles (Figure 8). Although discussed for many years,^[^ ^193^ ^]^ definitive proof of the involvement of covalent bonds in the extremely strong binding in primary aggregates was not established until recently, when spectroscopic studies showed that ether (C─O─C) bonds are present as links between the individual particles.^[^ ^194^ ^]^ The surface groups and the shape of the crystallites are responsible for the internal structure of the agglomerates,^[^ ^195^, ^196^, ^197^ ^]^ as the interplay between the localized charge on the functional groups, the polarity of the surface itself and the presence of nondiamond carbon govern the formation of lace‐like, fractal structures for negatively charged oxygenated nanodiamonds, whereas hydrogenated particles tend to form smaller, less hierarchical structures.^[^ ^198^ ^]^

In general, the formation of aggregates is governed by the surface termination, the morphology and size of the diamond particles. Not only can hydrogen bonds and electrostatic interactions lead to complex aggregate structures (Figure 8),^[^ ^198^, ^199^, ^200^, ^201^, ^202^ ^]^ but also the formation of covalent bonds has been shown.^[^ ^194^ ^]^ These include esters and ethers.^[^ ^194^, ^202^ ^]^ The resulting structures of these aggregates have been described as fractal^[^ ^203^ ^]^ and the formation of highly viscous hydrogels has been observed at elevated particle concentrations.^[^ ^204^ ^]^ Inside the aggregates, a rather large pore volume remains, rendering nanodiamond aggregates very hygroscopic.^[^ ^205^, ^206^ ^]^ It was also shown that the water molecules surrounding the diamond particles form an unusual network of hydrogen bonds, which contributes as well to the observed colloidal behavior.^[^ ^207^, ^208^ ^]^ Colloidal stability is influenced also by the concentration of the particles in the dispersion, but ionic impurities as well as nondiamond carbon on the surface play a role as well.^[^ ^193^, ^194^, ^205^, ^206^ ^]^

The adsorption of different types of small and large molecules can enhance the colloidal stability of nanodiamond in aqueous as well as in organic solvent environments. Dispersion is either achieved by electrostatic, steric, or electrosteric stabilization. The applied adsorbates include polymers, polymethine dyes, oxalic or glutamic acid, and propylamine, among many others.^[^ ^209^, ^210^, ^211^, ^212^ ^]^

#### 2.3.1 Synthesis of (Bio)Hybrid Materials

Bio‐applications of diamond particles come with several requirements regarding surface properties that must be met to ensure reproducible results. In most cases, it is necessary to obtain a stable colloidal dispersion of the particles in highly demanding physiological environments, i.e., containing high ion concentrations and a broad range of proteins and other cellular components. Several approaches have been investigated, including coating with polyglycerol,^[^ ^213^ ^]^ different variants of polyethylene glycol (PEG),^[^ ^214^, ^215^ ^]^ certain proteins^[^ ^216^ ^]^ or smaller structures such as zwitterionic moieties or amino acids.^[^ ^217^, ^218^, ^219^ ^]^ Another important aspect is the control of the interactions of the particle surface with the surrounding medium and, e.g., targeted areas. Here, the prevention of nonspecific interactions has been in the focus: The nature of the so‐called protein corona plays a major role in the interaction of nanodiamond with its environment^[^ ^220^, ^221^ ^]^ and thus its formation needs to be controlled. This has been achieved by immobilization of zwitterionic moieties,^[^ ^217^, ^218^, ^219^ ^]^ saccharides,^[^ ^222^, ^223^ ^]^ and tailored polymer coatings.^[^ ^214^, ^224^, ^225^, ^226^ ^]^

While many applications make use of the high stability of diamond's covalent surface bonds, diamond nanoparticles can also be used as carriers for intentional release and/or delivery of molecules of interest. Loading of nanodiamonds with drug molecules allows the efficient delivery of these drugs to different locations, such as tumors.^[^ ^227^, ^228^ ^]^ Here, both covalent binding and noncovalent coating have been used for efficient immobilization of drugs.^[^ ^229^ ^]^

Furthermore, the conjugation of functional diamond particles, e.g., with color centers or other intrinsic labels, with chromophores, sensors, or magnetic labels and spin probes has been used to provide multifunctional materials for biomedical applications. For example, the immobilization of dipeptides or gadolinium complexes allows for controlled interaction with physiological environments or multimodal MRI imaging, respectively.^[^ ^107^, ^173^ ^]^

Another important application is the delivery of microRNA or siRNA using nanodiamond as the vehicle.^[^ ^230^, ^231^, ^232^ ^]^ Here, the biocompatibility and the ability for tailored surface conjugation of complex biomolecules without altering their functionality are among the main benefits of nanodiamond application.

## 3 Emerging Areas and Future Opportunities for Diamond Chemistry

Advances in diamond growth have greatly improved the quality and purity of diamond materials that are available while reducing their cost. These advances have enabled a wide range of new applications of diamond, many of which have strong connections to chemistry and biology. Examples include electrochemical synthesis and electrochemical energy storage, environmental remediation, chemical and biological sensing, and biomedical applications such as drug delivery. Many of these applications have specific demands not only for the properties of the diamond bulk but also on the surfaces to control factors such as charge state, near‐surface conductivity, chemical reaction rates, electron‐transfer rates, and biomolecular recognition properties. Common to all applications is a need for highly stable and reproducible surface chemistry, extending from the fundamental chemistry of diamond growth and doping all the way to biomolecular recognition and use of active nanoparticles that, for example, can transform upon demand to deliver drugs or other reagents.

Below we briefly summarize some of the key challenges and opportunities in selected application areas as examples of some specific areas where greater understanding of diamond chemistry can have substantial impact.

### 3.1 Diamond Applications in Energy Storage

In recent studies, the construction of battery‐like diamond supercapacitors and diamond supercapbatteries that feature both high power and energy densities has been explored using nanostructured diamond electrodes and confined redox electrolytes. These and other energy storage systems taking advantage of nanostructured diamond electrodes have promise for electrochemical energy storage. For example, trapping inert/redox‐active electrolytes inside their pores/channels to form diamond SCs or diamond supercapbatteries has the potential to yield storage devices with both high energy densities and high power densities. Yet, much remains to be learned about the fundamental mechanisms and processes associated with ion adsorption and diffusion in such confined geometries. The chemical functionalization schemes that have been used successfully to control diamond surfaces in planar and nanoparticle form have yet to be explored or applied to confined geometries associated with these devices. The application of *in operando* analysis and computational modeling to provide insights into the thermodynamics and kinetics associated with these structures could guide development of structures and surface modifications able to take full advantage of the unique properties of diamond materials.

### 3.2 Catalysis and Electrocatalysis at Diamond Surfaces

Diamond materials have proven to be extremely attractive for several electrocatalytic and electrosynthesis processes, including selective electrochemical reduction of carbon dioxide into value‐added chemicals and liquid fuels, electrochemical nitrogen fixation, and electrochemical nitrite reduction into ammonia—an important industrial feedstock. For example, they show unique chemistry toward selective electrochemical energy conversion (e.g., CO_2_ reduction, nitrogen fixation) and electrosynthesis. Nanostructured diamond electrodes (e.g., diamond nanowires, porous diamond films, diamond particle‐based electrodes) will further improve their application performance.

However, opportunities for improvement through surface modification and functionalization of both the stability and rates of reaction lie ahead. Substrates that are co‐doped with boron + nitrogen or boron + phosphorus may provide multiple surface sites for reactions. Similarly, diamond composite materials may prove more effective than diamond alone. In situ identification and quantification of the generated radical and nonradical species or reaction pathways of these (photo)electrocatalytic reactions on diamond electrodes, especially when magnetic fields are further applied need to be carried out using advanced electron microscopy, operando electrochemical techniques, and computational calculations.

### 3.3 Environmental Remediation

Emerging concerns over perfluorinated alkyl substances (PFAS), compounds in groundwater and other places in the environment, have heightened interest in developing improved approaches to environmental systems.^[^ ^233^, ^234^, ^235^ ^]^ Electrochemical reduction of halogenated arenes and PFAS may be especially effective at diamond‐based electrodes due to diamond's large overpotential for H^+^ reduction and diamond's ability to directly inject electrons into aqueous media. Nanostructuring of the electrodes provides one pathway to achieving highly reactive diamond surfaces.^[^ ^154^ ^]^ The removal of highly problematic perfluorinated compounds such as perfluorooctanoic acid (PFOA) has been shown to be feasible with thermally annealed nanodiamond in the presence of peracetic acid or under UV irradiation in the presence of a polycrystalline diamond film.^[^ ^236^, ^237^ ^]^ The presence of surface defects and nondiamond carbon at the surface of the diamond has been suggested to be essential for the successful defluorination. In general, the generation of highly aggressive reductive or oxidative species is of importance for the decomposition of highly persistent pollutants.^[^ ^238^ ^]^ The generation of OH radicals, other reactive oxygen species (ROS), and even solvated electrons has been recently demonstrated.^[^ ^239^, ^240^ ^]^ A key challenge is that most of these processes are purely electrochemical. While diamond electrodes provide greater Faradaic efficiency compared to metallic electrodes, there are significant opportunities for improvement in selectivity and scalability. Since these reactions are all driven by processes at or near the electrode surfaces, nanostructuring of electrodes to provide higher surface areas could provide significant benefit. Many past studies of highly nonequilibrium reactions (e.g., CO_2_ reduction) have benefited from the use of surface‐tethered catalysts that incorporate rare and expensive transition metals (e.g., Ru).^[^ ^159^, ^160^ ^]^ Surface termination using benign moieties such as simple organic sensitizers or directly terminated diamond, preventing the use of critical transition‐metal‐based surface groups, would increase the overall sustainability of such approaches.

### 3.4 Diamond for Electron‐Induced Photoelectrochemistry

A more recent application of diamond as an electron emitter in water, air, and other nonvacuum environments presents new opportunities for photocatalysis and electrocatalysis.^[^ ^147^, ^148^, ^151^, ^152^, ^153^ ^]^ When terminated with H atoms, diamond's negative electron affinity allows it to be used as a unique electron emitter in nonvacuum environments, including water. As noted above, the emission of electrons into water produces highly reactive intermediates that can induce novel chemistry in the aqueous phase. However, reactive radical species such as H (the hydrogen atom) and OH (hydroxy radical) abstract H atoms from the H‐terminated surface, leaving it susceptible to oxidation by water. Functionalization of the surface with primary amino groups has been explored as an alternative termination providing a positively charged surface that is more resistant to oxidation than the H‐terminated surface.^[^ ^150^ ^]^ Amino (─NH_2_) groups are also typically protonated to ─NH_3_^+^ groups at ambient pH, further aiding electron emission. However, producing surfaces with a high concentration of amino groups remains a frontier area of diamond research, as gas‐phase plasma environments strongly favor N_2_ and H_2_ over NH*_x_* species. Recent studies have shown that photoelectrochemical properties may be retained even after surface oxidation,^[^ ^241^ ^]^ suggesting that requirements for surface termination may be different for electron emission into water compared to emission into vacuum. A second future direction is to make the electron emission more efficient when using longer‐wavelength (i.e., sub‐bandgap) sources. As in Figure 6, electron emission is typically initiated using short‐wavelength sources that excite across the bandgap (requiring light <225 nm wavelength). However, some emission is observed using sub‐bandgap sources.^[^ ^41^, ^149^, ^154^ ^]^ The mechanisms of sub‐bandgap emission have not yet been fully identified but may include direct excitation to high‐lying gap surface states or excitations involving impurities such as sp^2^‐hybridized (“graphitic”) carbon or other defects in the diamond lattice.^[^ ^149^, ^154^ ^]^ Recent studies have shown that diamond's electron emissive properties can also be enhanced by embedding silver nanoparticles into the film.^[^ ^242^ ^]^ Current and future research in this field would benefit by examining new approaches to enhancing sub‐bandgap electron emission pathways, such as the use of co‐catalysts.^[^ ^242^ ^]^ Similarly, efficiency could be improved by manipulation of diamond's optical properties through the introduction of various types of plasmonic nanoparticles into diamond films or through growth of novel heterostructures.^[^ ^78^, ^79^, ^80^ ^]^ While localized regions of sp^2^‐hybridized carbon can provide mid‐gap states that facilitate excitation to emissive surface states, they can also serve as recombination centers.^[^ ^154^ ^]^ Thus, understanding how the detailed dynamics of electron excitation and emission are influenced by different types of defects and/or heterostructures could be a promising area. Overall, continued research is needed to identify the best ways to manipulate diamond's optical and electron transport properties to control its photoelectrochemistry.

### 3.5 Diamond for Quantum Applications

**Figure 9:**
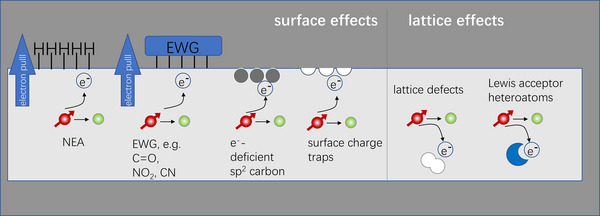
A large variety of parameters influence the charge state stability of NV centers in diamond. Adapted from Ref. [243].

As discussed earlier, the use of NV centers, SiV centers, and other types of quantum defects as highly sensitive readouts of local physical and chemical environments demands a high level of control over the diamond surfaces and near‐surface region. While NV centers and related color centers can be useful as simple optical fluorophores, many of the most exciting applications of diamond rely on the fact that these defects have unique spin properties that enable new modalities of chemical and biological sensing based on spin–spin interactions between the NV centers and nearby chemical or biological species of interest. Imperfections such as lattice defects and surface defects are primary sources of decoherence of NV‐based quantum states (Figure 9),^[^ ^243^ ^]^ thereby reducing the sensitivity and selectivity of the NV centers as local probes.

**Figure 10:**
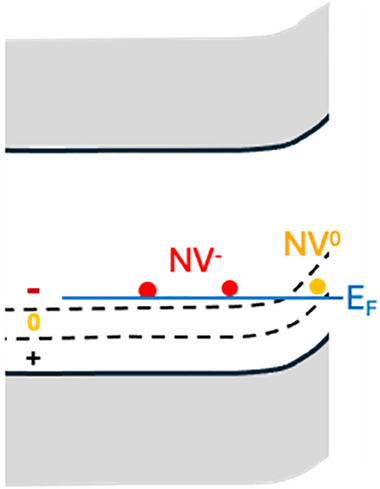
Influence of band‐bending on the charge state of NV centers. NV centers have three possible charge states with different regions of electrochemical stability as depicted. Upward band‐bending near the surface favors the NV° charge state over the NV^−^ charge state. As a result, NV centers can be in the (desirable) NV^−^ state in the bulk but in the (undesired) NV^0^ state near the surface.

Imperfections at the surface and in the near‐surface region adversely impact NV centers in two ways. First, unterminated surface chemical bonds, other surface defects, and paramagnetic surface contaminants introduce spin noise that decreases sensitivity.^[^ ^244^ ^]^ Second, the presence of electrically charged surface groups such as deprotonated surface hydroxyl groups or carboxylic acid groups alters the relative number of NV centers in the NV^0^ versus NV^−^ charge state, as depicted in Figure 10. Because these different charge states have different optical properties, uncontrolled interconversion of the charge states leads to uncontrolled alterations in sensitivity when used as optical readouts. Thus, the practical utilization of NV centers for sensing applications requires a high level of control over all types of inhomogeneities that impact the spin state or the charge state.

Some of the key challenges in this area include (1) how to terminate diamond surfaces in a way that maximizes the number of near‐surface NV centers in the desired charge state and (2) reducing or eliminating unterminated surface sites with unpaired electrons that contribute to spin noise.^[^ ^92^, ^185^ ^]^ Termination with H atoms generally favors the less desirable, neutral NV° center.^[^ ^137^ ^]^ Terminating with groups such as oxygen, nitrogen, or fluorine favors the NV^−^ center, but the larger size of these surface groups coupled with the small distance between surface carbon atoms makes perfect surface termination more difficult, leading to more spin noise from surface defects.^[^ ^166^, ^245^, ^246^, ^247^ ^]^ Recent computational studies have suggested that a surface termination containing a tailored mixture of ─H, ─OH, and C─O─C (ether) linkages may provide the optimal benefit.^[^ ^184^, ^185^, ^248^ ^]^ Yet, reproducibly preparing a surface with complex mixtures of terminations has not yet been achieved. Controlling the surface termination for diamond‐based applications remains a frontier area of research.

### 3.6 Biomedical Applications

Regarding biomedical applications, it becomes clear that the surface chemistry plays a significant role in the outcome of biological experiments. It has been shown that the cytotoxicity of diamond nanomaterials depends on the actual surface termination and the presence of nondiamond carbon.^[^ ^168^, ^249^, ^250^ ^]^ Therefore, for all biomedical investigations, it is required to fully characterize the surface and to undertake thorough efforts to provide reproducible and efficient surface purification and termination protocols. Additionally, the origin of the diamond nanomaterials plays a role in the actual properties. Nanoparticles of comparable size but different production methods show significantly distinct properties, such as rounded versus faceted surface morphologies, amount of surface‐bound nondiamond carbon and intrinsic nondiamond carbon embedded in the diamond lattice and a different portfolio of surface groups.^[^ ^251^ ^]^ In the future, standardized and regulated diamond resources for biomedical research will be required to advance in the field of clinical applications.

For biomedical applications, also beyond quantum sensing, the control of the surface is the main requirement: Not only do we need to develop even better methods for the synthesis of highly homogeneous diamond materials that interact with their surroundings in a highly defined manner, but also we need to understand the underlying mechanisms for the uptake and release of functionalized nanodiamond into and from biological entities. Only with full control of these processes and the knowledge of the influence of the surface structure, we will be able to leverage the full potential of nanodiamond. To this end, the formation of conjugates with defined stoichiometry is highly sought after. In the future, template‐assisted approaches or methods based on single‐site functionalized polymer coatings could open new opportunities in this regard. Furthermore, an in‐depth understanding of the influence of different material properties on the toxicity profile needs to be obtained to enable progress toward actual medical applications.

## 4 Summary

Diamond's emergence as a material with myriad applications in chemistry and biology provides a wealth of opportunities for chemists, both to play a role in the development and improvement of the chemistry of diamond growth and modification and in the expansion of applications of diamond in the chemical sciences. While we are not able to fully explore all chemically relevant applications of diamond in this perspective, the discussion above highlights some representative examples in areas of current and future relevance to the chemistry and biology communities.

## Conflict of Interests

The authors declare no conflict of interest.

## Data Availability

Research data are not shared.
